# Age-related changes of right atrial morphology and inflow pattern assessed using 4D flow cardiovascular magnetic resonance: results of a population-based study

**DOI:** 10.1186/s12968-018-0456-9

**Published:** 2018-06-14

**Authors:** Thomas Wehrum, Thomas Lodemann, Paul Hagenlocher, Judith Stuplich, Ba Thanh Truc Ngo, Sebastian Grundmann, Anja Hennemuth, Jürgen Hennig, Andreas Harloff

**Affiliations:** 1 Department of Neurology and Neuroscience, Medical Center - University of Freiburg, Faculty of Medicine, University of Freiburg, Breisacher Straße 64, 79106 Freiburg, Germany; 2 Department of Cardiology, University Heart Center Freiburg, Faculty of Medicine, University of Freiburg, Freiburg, Germany; 3 Charité – Universitätsmedizin Berlin, Institute for Imaging Science and Computational Modelling in Cardiovascular Medicine, Berlin, Germany; 4 Department of Diagnostic Radiology – Medical Physics, Medical Center, University of Freiburg, Faculty of Medicine, University of Freiburg, Freiburg, Germany

**Keywords:** Aging, Heart failure, Pulmonary hypertension, Magnetic resonance imaging (MRI)

## Abstract

**Background:**

To assess age-related changes of blood flow and geometry of the caval veins and right atrium (RA) using 4D flow cardiovascular magnetic resonance (CMR) data obtained in a population-based study.

**Methods:**

An age-stratified sample (*n* = 126) of the population of the city of Freiburg, Germany, underwent transthoracic echocardiography and electrocardiogram-triggered and navigator-gated 4D flow CMR at 3 Tesla covering the caval veins and right heart. Study participants were divided into three age groups (1:20–39; 2:40–59; and 3:60–80 years of age). Analysis planes were placed in the superior and inferior caval vein. Subsequently, RA morphology and three-dimensional blood inflow pattern was assessed.

**Results:**

Blood flow of the RA showed a clockwise rotating helix without signs of turbulence in younger subjects. By contrast, such rotation was absent in 12 subjects of group 3 and turbulences were significantly more frequent (*p* < 0.001). We observed an age-related shift of the caval vein axis. While the outlets of the superior and inferior caval veins were facing each other in group 1, lateralization occurred in older subjects (*p* < 0.001). A convergence of axes was observed from lateral view with facing axes in older subjects (*p* = 0.004). Finally, mean and peak systolic blood flow in the caval veins decreased with age (group 3 < 2 < 1).

**Conclusions:**

We have provided reference values of 4D CMR blood flow for different age groups and demonstrated the significant impact of age on hemodynamics of the RA inflow tract. This effect of aging should be taken into account when assessing pathologic conditions of the heart in the future.

**Electronic supplementary material:**

The online version of this article (10.1186/s12968-018-0456-9) contains supplementary material, which is available to authorized users.

## Background

Impaired venous return and right-heart function can be caused by a variety of pathologic conditions such as heart-failure, pulmonary hypertension, tricuspid valve disease, arrhythmogenic right ventricular (RV) cardiomyopathy and congenital heart disease [1–3]. However, the likelihood of development of right heart failure is increased in elderly patients independent of the underlying disease [4]. The specific reasons for this age dependency remain unknown so far. The main diagnostic method used for monitoring right-heart pressures is the pulmonary floatation catheter [5]. However, this technique is an invasive procedure which requires a central venous access and can lead to complications such as pneumothorax and hemothorax, arterial laceration, cardiac arrhythmias, valvular damage, pulmonary artery perforation, false aneurysms, and catheter knotting in the case of right-heart catheterization [6]. A non-invasive alternative for assessment of morphological and functional aspects of the caval veins and right heart is 2D transthoracic echocardiography (TTE). Unfortunately, TTE accuracy is limited by the complex crescent shaped RV geometry and by its location behind the sternum [7]. While 3D-TTE can overcome some of these restraints, its applicability is often limited by suboptimal acoustic windows. This limitation can be overcome by using 3D imaging techniques such as 3D cardiovascular magnetic resonance (CMR) which can improve assessment of morphological features [8]. Furthermore, application of flow-sensitive 3D phase-contrast CMR (4D flow CMR) allows in-vivo visualization of blood flow from the caval veins through the right atrium (RA) into the RV [9]. 4D flow CMR was already used to highlight the role of vortex formation in healthy individuals as a driving force of RA filling [10]. Furthermore, it can be used to evaluate indices of RV systolic function from volume segmentation [11]. Despite its inherent potential to comprehensively assess right heart filling and associated pathologic conditions, most applications of cardiac 4D flow CMR, however, have focused on the left heart [12, 13] or the pulmonary arteries [11, 14].

One major reason for this may be the lack of age stratified reference data for 4D flow CMR, which hamper interpretation of hemodynamic findings. Therefore, it was our aim to assess age-related morphological and functional changes of the RV inflow tract using 4D flow CMR data obtained in an age-stratified population and to provide reference values for future clinical applications.

## Methods

### Study population

We performed a cross-sectional observational study of an age-stratified sample of the population of the city of Freiburg, Germany (for details see [14]). The cohort was established on the basis of data obtained from the German Civil Registration System. We used six blocks of 20 subjects (ten female and ten male) with the following age-intervals: 20–29, 30–39, 40–49, 50–59, 60–69, and 70–80 years. Starting in October 2012, 3500 age-stratified and randomly selected registered residents of Freiburg were contacted by mail, asked to participate in our study, and provided with details on how to contact the study team. Three hundred eight subjects responded to our mail and were contacted by phone on the basis of first-come, first-served. One hundred forty-seven were excluded because of known CMR contraindications, too many participants in this group of age, or because of scheduling difficulties. One hundred sixty-one subjects were finally scheduled for CMR. In 23 subjects the CMR protocol was not completed for technical reasons, 11 were absent on the appointed day, 11 aborted the CMR examination because of claustrophobia, and five were not suitable for CMR due to contraindications. Because of insufficient response within the group of 20–29 year olds, the study was advertised on the University Hospital Freiburg intranet and persons within this age-interval were invited to participate. The first 16 persons who contacted the study team by email were included. One person had to be excluded for technical difficulties during CMR and one subject was absent on the appointed day. Finally, datasets of 126 subjects were available for data analysis. Cardiovascular risk factors and demographics were determined by interview. The study was approved by the University of Freiburg ethics committee (IRB number 227/14) and written informed consent was obtained from all participants.

### Transthoracic echocardiography

All participants underwent additional TTE using a Toshiba Artida system (4.8-2MHz PST-30BT transducer; Toshiba Medical Systems Corporation, Tokyo, Japan) based on the recommendations and standards of the American Society of Echocardiography [15]. Median time between CMR and TTE was 0 days (inter quartile range: 0–1).

### 4D flow CMR imaging

CMR examinations were conducted on a routine clinical 3 Tesla CMR system (TIM Trio, Siemens Healthineers, Erlangen, Germany), using a standard 12-element body coil. 4D flow CMR was acquired to obtain time-resolved and three-dimensional blood flow parameters of the caval veins, the RA, and the RV. All experiments were electrocardiogram (ECG)-synchronized as well as respiration-controlled and used navigator-gating to allow free breathing [16]. All datasets were acquired in end-expiration with the navigator placed on the liver dome in order to minimize motion artefacts induced by respiration. Parameters of 4D flow CMR were: echo time/repetition time = 2.6/5.1 ms, flip angle = 7°, temporal resolution = 20.4 ms, matrix size = 320 × 240 × 58, bandwidth = 450 Hz/pixel, spatial resolution = 2.1 × 2.1 × 2.5 mm^3^, velocity sensitivity along all three directions = 150 cm/s, and parallel imaging (PEAK-GRAPPA) along the phase encoding direction (y) with an acceleration factor of *R* = 5 (20 reference lines).

### CMR data analysis

**Fig. 1:**
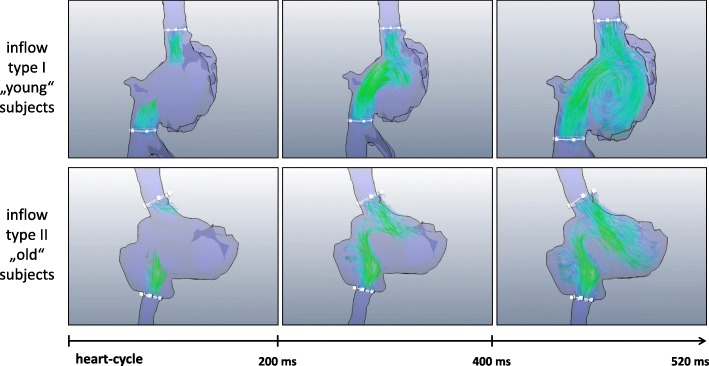
Visualization of atrial filling. Visualization of right atrial filling showed two patterns of inflow: In young subjects, formation of a clockwise-rotating vortex was observed. In older subjects, atrial filling was more turbulent and vortex formation was even absent in some subjects

**Fig. 2:**
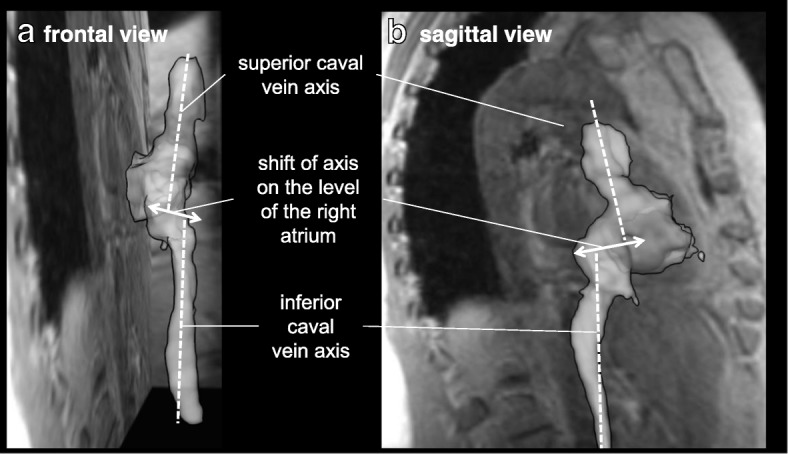
Measurement of axis-shift. Measurement of axis-shift of the superior and inferior caval vein in (**a**) frontal and (**b**) lateral view. Two axes through the centers of the superior vena cava (SVC) and inferior vena cava (IVVC) lumen were manually defined and the shift of axes was measured in sagittal and frontal view in cm

CMR data were analysed using MEVISFlow software (Fraunhofer MEVIS, Bremen, Germany) (for technical details see [17]). After corrections for eddy-currents and phase-wraps, and vessel-segmentation, analysis planes were positioned perpendicular to the vessel lumen in the superior vena cava vein (SVC), the inferior vena cava (IVC), and in the ascending aorta to measure cardiac stroke volume. The SVC and IVC planes were located with a distance of 1 cm from the entry to the RA to account for flow turbulences in this area (see Fig. 1). For each analysis plane, a lumen contour surrounding the lumen had to be defined manually while the contour was adapted automatically to all time points [17] and flow was visualized simultaneously in the SVC and IVC plane. Flow analysis comprised the following parts:

1. The visual pattern of RA inflow was studied based on movies of individual 3D blood flow (Additional file 1: Video S1) that could be contemplated from different views. A blinded observer with experience in 4D flow CMR data analysis of 6 years performed the assessment of the data. The right-heart was analyzed from a lateral and a frontal point-of-view and the appearance of RV inflow was graded as 1: appearance of vortex with clockwise rotation, 2: appearance of vortex with counter-clockwise rotation, and 3: no visible rotation.
2. Furthermore, the geometry of the RV inflow tract was analyzed on 3D vessel segmentations obtained during 4D flow CMR. Two axes through the centers of the SVC and IVC lumen were manually defined and the shift of axes was measured in sagittal and frontal view in cm (see Fig. 2). Measurements were made to the nearest whole centimetre.
3. Flow volumes in the SVC, IVC, and ascending aorta analysis plane were quantified for every time step.

### Measurement of individual right atrial volume

One investigator who was blinded to patient data performed area measurements of the RA in four-chamber view and right-sided two-chamber view utilizing retrospectively gated 3D phase contrast CMR images which were acquired in end-expiration. Impax software (Agfa Healthcare, Bonn, Germany) was used for area measurements. The RA appendage was not included in the measurements. The equation (RA volume = 3.08 * (2C area) + 3.36 * (4C area) − 44.4) [18] was previously used to calculate right atrial volume. All areas derived from 2D images were measured in the phase at which the RA size was at a maximum, i.e. during ventricular systole. Body surface area (BSA) was calculated according to the Mosteller formula [19]. RA volume was indexed to BSA to obtain the right atrial volume indexed to BSA (RAVI).

### Statistical analysis

Data are presented as mean ± standard deviations (SD) or median (interquartile range) for continuous, absolute, and relative frequencies for categorical variables. Patients were categorized according to their age into three groups which were used for comparisons of planar blood flow, inflow visualization, and geometry of the inflow tract: group 1 (20–39 years), group 2 (40–59 years), and group 3 (60–80 years). The TTE parameter dPmax RVRA which represents the maximum pressure gradient between the RV and the RA was used as a surrogate of pulmonary arterial (PA) systolic pressure and was used to test for an association between elevated PA pressure and occurrence of morphological changes and changes of the RA inflow pattern. Furthermore, we tested whether presence of tricuspid regurgitation influences the studied parameters. Departures from normality were detected with the Shapiro-Wilk statistic. Homogeneity of variance was assessed using Levene’s test. Differences between patient groups were evaluated using Fisher’s exact test and one-way ANOVA with Tukey’s HSD post-hoc test. All tests were two-sided with 0.05 as the level of statistical significance. Statistical analyses were performed using SPSS (Statistics version 19.0.1. International Business Machines, Armonk, New York, USA)

## Results

### Patient characteristics

Cardiovascular disease risk factors and patient demographics are presented in Table 1. Hypertension and hypercholesterolemia occurred more often in older subjects (group 3 > 2 > 1) while no differences between groups were observed regarding other risk factors. Only few subjects had diabetes (*n* = 2), prior stroke (*n* = 2), coronary-artery-disease (*n* = 2), and none of the subjects suffered from peripheral vascular disease.

**Table 1:** Demographics and cardiovascular risk factors of study participants

Characteristics of patients	Group 1	Group 2	Group 3	*p*-value
	(20–39 years)	(40–59 years)	(60–80 years)	
	*n* = 43	*n* = 44	*n* = 39	
Age, years ± SD	30.1 ± 5.4	50.4 ± 5.5	68.9 ± 5.2	< 0.001*
Female, *n* (%)	19 (44.2)	24 (54.6)	21 (53.9)	0.564
Hypertension, *n* (%)	1 (2.3)	7 (16.3)	13 (33.3)	< 0.001*
Hypercholesterolemia, *n* (%)	1 (2.3)	9 (20.5)	11 (28.2)	0.005*
Diabetes, *n* (%)	0 (0.0)	1 (2.3)	1 (2.6)	0.587
Smoker, *n* (%)	11 (25.6)	6 (13.6)	5 (12.8)	0.223
Body mass index, 1 ± SD	24.0 ± 4.0	26.0 ± 4.4	24.31 ± 3.8	0.051
Prior stroke, *n* (%)	0 (0.0)	2 (4.6)	0 (0.0)	0.151
Coronary arterydisease, *n* (%)	0 (0.0)	0 (0.0)	2 (5.1)	0.104
Peripheral arterial disease, *n* (%)	0 (0.0)	0 (0.0)	0 (0.0)	–
Mean systolic blood pressure, mmHg ± SD	120.6 ± 11.1	126 ± 15.7	133.8 ± 19.2	< 0.001*
Mean diastolic blood pressure, mmHg ± SD	76.5 ± 7.0	81.9 ± 8.3	81.1 ± 11.4	0.014*
Heart rate, bpm ± SD	67.2 ± 7.9	65.6 ± 7.7	66.1 ± 9.1	0.655

Values are given as absolute values (percentage) or ± *SD* = standard deviation, *bpm* beats per minute; *Significant

### Transthoracic echocardiography (TTE)

Results of TTE are displayed in Table 2. None of the subjects had significant tricuspid and/or pulmonary valve pathologies (regurgitation grade III or IV) and/or reduced ejection fraction. We observed a small trend towards a higher prevalence of the presence of tricuspid valve regurgitation grade I or II in the elder study group (group 3: *n* = 13 (33.3%) vs. group 2: *n* = 4 (9.1%) vs. group 1: *n* = 6 (13.9%); *p* = 0.019). Furthermore, maximum pressure difference between the RV and RA increased significantly from group 1 = 15.8 ± 2.4, group 2 = 18.2 ± 4.8, to group 3 = 22.1 ± 5.7 (*p* < 0.001). However, no significant trends were observed regarding other RA TTE parameters.

**Table 2:** Results from transthoracic echocardiography of study participants

Characteristics of patients	Group 1	Group 2	Group 3	*p*-value
	(20–39 years)	(40–59 years)	(60–80 years)	
	*n* = 43	*n* = 44	*n* = 39	
Ejection fraction, % ± SD	56.6 ± 20.7	57.6 ± 19.2	53.6 ± 22.5	0.669
IVC collapse not complete, *n* (%)	1 (2.3)	1 (2.3)	0 (0.0)	0.595
PR grade I or II, *n* (%)	3 (7.1)	1 (2.3)	1 (2.6)	0.246
PR grade III or IV, *n* (%)	0 (0.0)	0 (0.0)	0 (0.0)	n/a
TR grade I or II, *n* (%)	6 (13.9)	4 (9.1)	13 (33.3)	0.019*
TR grade III or IV, *n* (%)	0 (0.0)	0 (0.0)	0 (0.0)	n/a
TAPSE, cm ± SD	22.9 ± 4.1	22.6 ± 3.3	23.1 ± 3.7	0.821
TVS’, cm/s ± SD	15.5 ± 2.9	15.53 ± 1.8	15.8 ± 1.8	0.847
dPmax MV, mmHg ± SD	3.5 ± 1.8	3.5 ± 1.3	3.6 ± 1.4	0.864
dPmax RVRA, mmHg ± SD	15.8 ± 2.4	18.2 ± 4.8	22.1 ± 5.7	< 0.001*

*EF* ejection fraction, *IVC* inferior vena cava, *PR* pulmonary egurgitation, *TR* tricuspid regurgitation, *TAPSE* tricuspid annular plane systolic excursion, *TVS’* tissue Doppler derived right ventricular systolic excursion velocity, *dPmax MV* maximum pressure gradient at the level of the mitral valve in diastole, *dPmax RVRA* maximum pressure at the level of the right ventricle/right atrium; *Significant

### Visualization of right atrial flow

In three datasets visualisation of flow was not possible due to technical reasons; those datasets were omitted in the following analysis. Pathline visualization of RA inflow showed a clockwise rotating helix without signs of turbulence in younger patients. Subjects in the group of 60–80 year olds had no visible rotation in 12 cases as compared to four and three participants in group 1 and 2, respectively (see Table 3.). The degree of visually observed turbulence was significantly higher in older subjects (*p* < 0.001).

**Table 3:** Prevalence of patterns of inflow and flow turbulence

	Group 1	Group 2	Group 3	All
	(20–39 years)	(40–59 years)	(60–80 years)	
	*n* = 43	*n* = 44	*n* = 39	*n* = 126
Inflow-morphology				
Clockwise	38 (88.4)	36 (81.8)	17 (43.6)	91 (72.2)
Counter-clockwise	1 (2.3)	4 (9.1)	8 (20.5)	13 (10.3)
No rotation	4 (9.3)	3 (6.8)	12 (30.8)	19 (15.1)
Flow turbulence				
No	37 (86.1)	17 (38.6)	7 (17.9)	61 (48.4)
Weak	2 (4.7)	8 (18.2)	5 (12.8)	15 (11.9)
Medium	1 (2.3)	12 (4.6)	9 (23.1)	22 (17.5)
Strong	3 (6.9)	6 (13.6)	16 (41.0)	25 (19.8)

### Geometry of the right ventricular inflow tract

Interestingly, an age related shift of the caval vein axis was observed (Fig. 1). Median (inter quartile range) frontal axis shift was 0 (0–0) cm for group 1, 1 (0–1) cm for group 2, 1 (0–2) cm for group 3, and lateral axis shift was 1 (1–2) cm for group 1, 1 (1–2) cm for group 2, and 1 (0–2) cm for group 3. While the outlets of the SVC and IVC were directly facing each other on frontal view in most young subjects, a lateralization was observed in older subjects which was most prominent in the group of 60–80 year olds (*p* < 0.001) (see Table 4.). Furthermore, a convergence of axes was observed from a lateral view and older subjects tended to have facing lateral axes (*p* = 0.004).

**Table 4:** Degree of frontal and lateral axis shift in study participants

	Group 1	Group 2	Group 3	All
	(20–39 years)	(40–59 years)	(60–80 years)	
	*n* = 43	*n* = 44	*n* = 39	*n* = 126
Frontal axis shift, *n* (%)				
0 cm	38 (88.4)	20 (45.5)	14 (35.9)	72 (57.1)
1 cm	2 (4.7)	19 (43.2)	14 (35.9)	35 (27.8)
2 cm	3 (6.9)	4 (9.1)	9 (23.1)	16 (12.7)
Lateral axis shift, *n* (%)				
0 cm	2 (4.7)	6 (13.6)	12 (30.8)	20 (15.9)
1 cm	28 (65.1)	25 (56.8)	11 (28.2)	64 (50.8)
2 cm	13 (30.2)	12 (27.3)	12 (30.8)	37 (29.4)
3 cm	0 (0.0)	0 (0.0)	2 (5.1)	2 (1.6)

### Age related change of caval flow

**Fig. 3:**
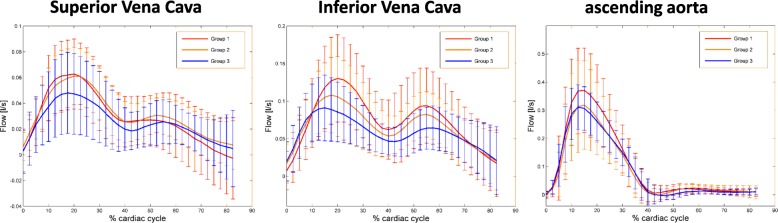
Caval vein and aortic flow profiles. The flow profile during the cardiac cycle is given for the SVC and IVC, and the ascending aorta. Groups are represented by different colors (red: group 1 (age 20–39); orange: group 2 (age 40–59); blue: group 3 (age 60–80))

Parameters of planar flow in the SVC and IVC are given in Table 5. RV inflow from the caval veins was lower in older patients (group 3 < 2 < 1), while the relative contribution of the single SVC and IVC to overall inflow remained constant (46–52%; *p* = 0.43). This finding is consistent with the change of stroke volume, which was also lower in older patients as measured in a standardized analysis plane of the ascending aorta (group 3 < 2 < 1), see Fig. 3. We did not detect differences between male and female subjects after adjustment for age and body-mass-index. The flow profile in the SVC and IVC showed flow peaks in systole and diastole (Fig. 3). Furthermore, blood flow reversal occurred in some subjects at the end of the heart cycle, which represented atrial systole. Peak systolic flow volumes were greater in younger patients in the SVC (group 1 > 2 > 3) and IVC (group 1 > 2 > 3), which was also true for peak diastolic flow in the ICV (group 1 > 2 > 3) but not in the SVC where no significant difference was observed (see Table 5).

**Table 5:** Planar flow volumes as measured in the superior (SCV), inferior (ICV), and both caval veins (CV)

	Group 1	Group 2	Group 3	F(2,375)	*p*-value
	(20–39 years)	(40–59 years)	(60–80 years)		
	*n* = 43	*n* = 44	*n* = 39		
Q_antegrade_, mL ± SD					
SVC	24.2 ± 6.4	24.5 ± 7.6	22.0 ± 6.8	1.6	0.210
IVC	57.2 ± 21.0	51.3 ± 18.4	45.8 ± 1.6	3.8	0.025*
CV	81.4 ± 23.3	75.9 ± 21.9	67.8 ± 17.8	4.2	0.017*
Q_retrograde_, mL ± SD					
SVC	0.5 ± 0.6	0.2 ± 0.3	0.2 ± 0.4	4.3	0.015*
IVC	0.4 ± 0.6	0.1 ± 0.3	0.1 ± 0.3	4.2	0.017*
CV	0.8 ± 1.1	0.4 ± 0.5	0.4 ± 0.5	5.9	0.004*
Q_ratio (SCV/ICV)_	0.5 ± 0.2	0.5 ± 0.2	0.5 ± 0.3	0.9	0.427
SV, mL/cycle ± SD					
AAo	82.2 ± 20.9	73.4 ± 15.6	66.5 ± 13.9	8.7	< 0.001*
CO, L/min ± SD	5.3 ± 1.6	4.8 ± 0.9	4.4 ± 1.1	6.1	0.003*
Qmax systole					
SVC	74.2 ± 24.2	68.8 ± 20.7	59.9 ± 23.4	4.1	0.02*
IVC	151.1 ± 55.3	119.8 ± 49.6	100.5 ± 40.7	3.7	0.02*
Qmax diastole					
SVC	36.4 ± 18.8	37.5 ± 12.3	31.5 ± 15.7	11.2	< 0.001*
IVC	109.1 ± 45.9	90.0 ± 31.2	71.3 ± 30.8	10.8	< 0.001*

*Qantegrade* flow rate (ml/min) in antegrade direction, *Qretrograde* flow rate (ml/min) in retrograde direction, *Qratio* ratio of total flow in SVC and IVC, *SV* stroke volume, *CO* cardiac output, *Qmax systole* maximum flow in systole, *Qmax diastole* maximum flow in diastole; *Significant

### Right atrium volume

RAVI was 25.2 ± 6.3 ml/m^2^ for group 1, 30.8 ± 9.4 ml/m^2^ for group 2, and 37.6 ± 9.4 ml/m^2^ for group 3. Thus, RAVI increased with age (*p* < 0.05 level for the three conditions [F(2,117) = 1509.7, *p* < 0.001]). In addition, we detected a trend of lower RAVI in subjects with a more shifted lateral caval vein axis [F(4,115) = 3.7, *p* = 0.007], and a higher RAVI in subjects with occurrence of RA turbulences [F(4,115) = 4.8, *p* = 0.001]. However, no association was visible for RAVI and the caval flow volumes, frontal axis shift, and flow morphology.

### Influence of pulmonary arterial pressure and tricuspid insufficiency

We did not detect significant differences of dPmax RVRA between patients with different inflow morphology (*p* = 0.181), turbulences (*p* = 0.089), and frontal axis shift (*p* = 0.145). However, dPmax RVRA was significantly lower in subjects with larger lateral caval vein axis shift [F(3,50) = 2.8, *p* = 0.047]: 0 cm: 22.7 ± 4.7 mmHg, 1 cm: 17.2 ± 4.5 mmHg, 2 cm: 18.5 ± 5.4 mmHg. When comparing the presence of tricuspid regurgitation with our studied parameters we did not find a significant association of the inflow morphology (*p* = 0.49), the occurrence of turbulences (*p* = 0.50), lateral axis shift (*p* = 0.78), frontal axis shift (*p* = 0.55).

## Discussion

We performed 4D flow CMR of the RA inflow tract and the SVC and IVC in an age-stratified sample of 126 subjects of the population of a medium sized city in southern Germany. This is the first study investigating morphological and functional changes of the RA inflow tract in a large sample of the general population, systematically covering all age groups. Our results indicate that blood flow to the heart decreases with age. This effect may be a result of declining cardiac output which in turn leads to decreased venous return to the heart. One recent study quantified RA filling in 12 healthy subjects (8 male; 40 ± 13 years) using 4D flow CMR. They found similar combined stroke volumes of the RA inflow tract with a total stroke volume of 76.6 ± 21.4 ml (SVC 18.1 ± 7.1 ml; IVC 58.9 ± 19.6 ml) [10], compared to 75.9 ± 21.9 ml (SVC 24.5 ± 7.63 ml, IVC 51.3 ± 18.35 ml) in the group of 40–59 year old subjects in our study. The same was true for one study examining 10 healthy subjects (6 male; mean age 30 years) in which median flow at end inspiration was 31.0 ml in the SVC and 56.1 ml in the IVC [20], as compared to 24.2 ± 6.39 ml for the SVC and 57.2 ± 21.01 ml for the IVC in the group of 20–39 year old subjects in our study. A further study investigating SVC and IVC blood flow in atrio-pulmonary anastomosis did show higher stroke volumes in the SVC (70.0 ± 30.0 ml) and IVC (180.0 ± 40.0 ml) [21]. However, the SVC to IVC ratio (~ 1:2) was similar to our and the aforementioned studies [10, 21]. Interestingly, the ratio between SVC and IVC did not differ between different age groups in our study.

Our results indicate that morphological and functional changes of RA venous filling take place in the aging heart. Many studies have focussed on the effects of aging on left ventricular and left atrial morphology and the occurrence of cardiac hypertrophy and atrial fibrillation. However, our study is the first to systematically investigate morphological changes of the RA inflow tract during aging in a large sample of the general population. One recent study (13 healthy subjects [6 male, mean age 40 years] and 13 subjects [6 male, 40 years] with cryptogenic stroke and patent foramen ovale) also assessed the position of the caval veins by comparing the points at which the centerlines of the respective caval vein flows intersected with the orthogonal planes placed at the junction with the RA, and found positions of the caval veins to be constant in the antero-posterior direction but significantly variable in the right-left direction [22]. In the right-left plane the separation between the IVC and the SVC was greater in the group of subjects with patent foramen ovale compared to controls (10 ± 5 mm versus 3 ± 3 mm, *p* = 0.002) [22], as compared to 8.6 mm in the group of 60–80 year olds in our study.

RA volume was larger in older patients in our study and may explain the phenomena reported in our study in particular given the fact, that the occurrence of turbulences and the lateral axis shift was associated with changes in atrial volume. Our results regarding RAVI are lower than reported in the literature (54 mL/m2 [95% CI: 34, 75]) [18]. All diameters and areas derived from 2D images in the literature [18] and in our study were measured in the phase at which the RA size and volume measurements were at a maximum, i.e. ventricular systole. In the literature, measurements of RA volumes [18] were performed using retrospectively ECG triggered balanced steady-state free precession imaging end-expiratory breath-hold cines which were acquired in the two (left and right chambers) and four chamber views, with subsequent contiguous short-axis cines from the atrioventricular ring to the base of the atria. This sequence was not included in our current study protocol and our measurements were based on a combination of the extracted vessel surface based on 3D phase contrast CMR datasets and a volume rendering of the magnitude image. This may have resulted in underestimation of the absolute RA volumes, however as this effect applies to all our study subjects it should not have influence on the association of RA volumes and the flow phenomena reported in this study.

We only detected a significant association between lateral caval vein axis shift and higher PA pressure with the parameter dPmax RVRA. However, our data demonstrate a trend that patients with elevated PA pressure also had more turbulent RA inflow, although the statistical level of significance was missed (*p* = 0.089) potentially due to the limited sample size. Furthermore, no subjects with pulmonary artery hypertension were included in our population based study. Accordingly, studying morphological and functional parameters of RA inflow using 4D flow CMR would be interesting in future studies investigating changes in patients with pulmonary artery hypertension.

Cardiac looping during embryonic development leads to asymmetries and curvatures which have potential fluidic and dynamic advantages [23]. This is expressed by the uniform clockwise rotating helix of blood flow in healthy subjects comprising 80% of ventricular inflow volume as shown in a previous study [10]. This mechanism allows the momentum of inflowing streams to be redirected towards atrio-ventricular valves. Interference with ventricular ejection flow may be minimized by the change in direction at the ventricular level which can enhance ventriculo-atrial coupling and may minimize dissipative interaction between entering, recirculating and outflowing streams [23]. These factors might combine to improve hemodynamics when heart rate and output increase during exercise. However, this mechanism may be weakened by morphological changes of the aging heart such as divergence of the frontal axis of the caval veins and convergence of the lateral axis. As a result, the convergence of inflowing streams of blood from the IVC and SVC occurs at a different angle, which impedes formation of a clockwise rotating helix and makes room for turbulences to occur. These turbulences and the loss of fluidic and dynamic advantages lead to diminished inflow and may further increase susceptibility for right heart failure in elderly patients. Furthermore, one recent study showed that absence of the ‘standard’ vortex during systole and diastole was more common in 13 subjects with patent foramen ovale and cryptogenic stroke compared to age-matched controls [22]. Hence, future large scale studies on cryptogenic stroke may be a potential application of vortex analysis using 4D flow CMR in the RA.

Respiratory activity affects venous return through changes in RA pressure and changes of the volume of the caval veins (e.g. vena cava compression) and cardiac chambers (e.g. changing cardiac preload). During expiration the chest wall collapses and the diaphragm ascends which makes the intrapleural pressure become more positive resulting in reduction of cardiac chamber and caval vein size. All datasets were acquired in end-expiration in this study. Accordingly, absolute flow values and volumes presented here may be smaller compared with data acquisition in end-inspiration. However, as all datasets were acquired similarly this has no influence on the analysis of age-related changes of RA morphology and filling.

Potential limitations of our study were the relative small sample size and the amount of initial nonresponse to our study invitation. When compared with registry data [24] and data from another German population-based study [25] the subjects under investigation less often had hypertension, hypercholesterolemia, diabetes, were less often smokers, obese, and only few patients suffered from a cardiovascular disease. This was probably due to the recruitment modality which required participants to actively contact the study team and to visit the University Medical Center without receiving a reimbursement. Accordingly, particularly healthier or health-conscious residents were probably interested in collaborating in this study. Inclusion of sufficient number of young (20–29 years of age) males is a common problem in cohorts like ours. Accordingly, 14 included subjects were recruited from the personnel of our institution (*n* = 922 male 20–29 years of age) on the basis of first-come, first-served to minimize bias.

## Conclusions

In conclusion, we have shown that 4D flow CMR can be used to visualize and assess flow in the caval veins and in the RA inflow tract. Furthermore, we provided reference values for 4D flow CMR based flow quantification in the caval veins. We demonstrated that age has a significant impact on caval blood flow and RV hemodynamics. This effect of aging should be taken into account when assessing pathologic conditions of the heart in future studies using 4D flow CMR. Findings of this population-based study are highly valuable for comparison with those in patients with manifest cardiac diseases.

## Additional file

Additional file 1: **Video S1.** Visualization of the typical right atrial inflow pattern. In most subjects a clockwise-rotating vortex was observed. (MP4 326 kb)

## Abbreviations

BP: Blood pressure
BSA: Body surface area
CMR: Cardiovascular magnetic resonance
dPmax RVRA: maximum pressure at the level of the right ventricle/right atrium
ECG: Electrocardiogram
GRAPPA: Generalized Autocalibrating Partially Parallel Acquisitions
IVC: Inferior vena cava
PA: Pulmonary artery
PR: Pulmonary regurgitation
RA: Right atrium/right atrial
RAVI: Right atrial volume index
RV: Right ventricle/right ventricular
SVC: Superior vena cava
TR: Tricuspid regurgitation
TTE: Transthoracic echocardiography
